# Supplementary material for: Deletion of wfs1 Impairs Oligodendrocyte Precursor Cells Dorsal Distribution and Myelination Through the wfs1-hmgcs1 Axis in Zebrafish
Source: Biology (Basel). 2026 Aug 16;15(16):1402. doi: 10.3390/biology15161402 (PMC13510151; doi:10.3390/biology15161402)
Supplement: Supplementary file 1 [file biology-15-01402-s001.zip › File S1-Transcriptome sequencing data and differentially expressed genes (DEGs) in wfs1-- zebrafish at 5 dpf/2.KEGG/wfs1KOvsWT/ALL/src/dre03015.html]

dre03015


- dre:563172

- Down regulated genes

563172(-1.66699314896147)

- dre:321035

- Up regulated genes

321035\_1(2.36627873946462)

- dre:562282

- Up regulated genes

562282(1.6446353235091)

- Down regulated genes

562282\_1(-5.61712483666314)
- dre:559392

- Up regulated genes

559392\_1(5.40322756218621)

- Down regulated genes

559392(-2.91317811245432)

- dre:321035

- Up regulated genes

321035\_1(2.36627873946462)

- dre:324267

- Up regulated genes

324267\_1(6.83370155064563)

- Down regulated genes

324267(-4.07498915678501)

- dre:100002680

- Up regulated genes

100002680\_2(2.08900246284662)

- dre:492341

- Down regulated genes

492341\_1(-4.29180794794591)

- dre:100005893

- Up regulated genes

100005893(2.69284130859211)

- Down regulated genes

100005893\_1(-1.31383589537958)

- dre:393693

- Up regulated genes

393693\_1(8.42626553180885)

- Down regulated genes

393693(-1.24516760321911)

- dre:394058

- Down regulated genes

394058\_1(-1.99218425808186)

- dre:386630

- Up regulated genes

386630\_1(7.78558729858034)

- Down regulated genes

386630(-5.68611972795874)

Close
